# Effect of prucalopride to improve time to gut function recovery following elective colorectal surgery: randomized clinical trial^[Author-notes znac121-FM1]^

**DOI:** 10.1093/bjs/znac121

**Published:** 2022-05-26

**Authors:** Tony Milne, Chen Liu, Greg O’Grady, John Woodfield, Ian Bissett

**Affiliations:** Department of Surgery, University of Auckland, Auckland, New Zealand; Department of Surgery, University of Auckland, Auckland, New Zealand; Department of Surgery, Auckland District Health Board, Auckland, New Zealand; Department of Surgery, Southern District Health Board, Auckland, New Zealand; Department of Surgery, Auckland District Health Board, Auckland, New Zealand

## Abstract

**Background:**

Delayed return to gut function and prolonged postoperative ileus (PPOI) delay recovery after colorectal surgery. Prucalopride is a selective serotonin-4-receptor agonist that may improve gut motility.

**Methods:**

This was a multicentre, double-blind, parallel, placebo-controlled randomized trial of 2 mg prucalopride *versus* placebo in patients undergoing elective colorectal resection. Patients with inflammatory bowel disease and planned ileostomy formation were excluded, but colostomy formation was allowed. The study medication was given 2 h before surgery and daily for up to 6 days after operation. The aim was to determine whether prucalopride improved return of gut function and reduced the incidence of PPOI. The primary endpoint was time to passage of stool and tolerance of diet (GI-2). Participants were allocated in a 1 : 1 ratio, in blocks of 10. Randomization was computer-generated. All study personnel, medical staff, and patients were blinded.

**Results:**

This study was completed between October 2017 and May 2020 at two tertiary hospitals in New Zealand. A total of 148 patients were randomized, 74 per arm. Demographic data were similar in the two groups. There was no difference in median time to GI-2 between prucalopride and placebo groups: 3.5 (i.q.r. 2–5) *versus* 4 (3–5) days respectively (*P* = 0.124). Prucalopride improved the median time to passage of stool (3 *versus* 4 days; *P* = 0.027) but not time to tolerance of diet (2 *versus* 2 days; *P* = 0.669) or median duration of hospital stay (4 *versus* 4 days; *P* = 0.929). In patients who underwent laparoscopic surgery (125, 84.5 per cent), prucalopride improved median time to GI-2: 3 (2–4) days *versus* 4 (3–5) days for placebo (*P* = 0.012). The rate of PPOI, complications, and adverse events was similar in the two groups.

**Conclusion:**

Prucalopride did not improve time to overall recovery of gut function after elective colorectal surgery. Registration number: NCT02947269 (http://www.clinicaltrials.gov).

## Teaser

This multicentre double-blind placebo-controlled RCT used prucalopride in patients undergoing elective colorectal surgery; 148 patients were recruited. Prucalopride did not improve overall time to return of gut function, but significantly improved time to postoperative gut recovery in patients who underwent laparoscopic surgery. Prucalopride was safe in this cohort.

## Introduction

Delayed return of gut motility after surgery, or postoperative ileus, is a predominant cause of delayed discharge after elective colorectal surgery^1^. When delayed gut motility persists for 4 or more days after operation, this is called prolonged postoperative ileus (PPOI)^2^. PPOI is one of the most common complications after major colorectal surgery. PPOI occurs in around 10–25 per cent of patients who undergo elective colonic or rectal surgery, and leads to significant patient morbidity and increased healthcare costs. More than 50 per cent of patients who develop PPOI develop a further complication^3^, and patients with PPOI spend twice as long in hospital^4^. The inpatient economic burden of PPOI is estimated to be an additional €7880 per patient affected with ileus^5^, or €1.4 billion per year in the USA alone^6^.

One of the key problems with delayed return to normal gut motility, and PPOI, is that there are few effective treatments, or preventative measures to reduce its burden^7^. Postoperative ileus affects the entire gastrointestinal tract^8,9^, meaning that prokinetics which only selectively target the colon or stomach, for example, are unlikely to be effective. Recent evidence suggests that the pathophysiology of postoperative ileus is mediated by an initial neurologically mediated phase and then a delayed inflammatory response to surgery in the gut, and that this inflammatory response can be diminished by drugs that potentiate vagal nerve activity^8–10^.The inflammatory phase is thought to contribute to the development and duration of PPOI^11^.

Prucalopride is a serotonin-4-receptor agonist that increases presynaptic acetylcholine release from vagal neurones, improves colonic motility, and is a gastric prokinetic^12–15^. A recent trial by Gong and colleagues^16^ showed that administration of prucalopride after elective colorectal surgery improved time to passage of flatus and stool. Recent studies have shown that prucalopride, when given before surgery and continued in the postoperative phase, reduces the inflammatory response associated with surgery^17^, and improves time to tolerance of diet^18^.

The primary aim of this double-blind placebo-controlled randomized trial was to investigate whether prucalopride administered before operation and continued afterwards improves time to return of postoperative gut motility in patients undergoing elective colorectal resection. The secondary aim was to determine whether prucalopride reduces the incidence of PPOI after elective colorectal surgery.

## Methods

The study was approved by the New Zealand Health and Disability Ethics Committee, as well as by the Auckland District Health Board and Southern District Health Board Research Review Committees. The trial was registered prospectively with ClinicalTrials.gov (NCT02947269).

### Study design

This multicentre, double-blind, parallel, placebo-controlled randomized trial was undertaken to investigate whether prucalopride improves time to recovery of postoperative gut function compared with placebo. Eligible patients were aged 18 years or older, who had elective colorectal surgery at one of two tertiary New Zealand hospitals (Auckland City Hospital and Dunedin City Hospital) between October 2017 and May 2020. Patients and the public were not involved in the design or conduct of this study. There was no change to the trial methods after commencement.

#### Inclusion criteria

Patients who underwent right hemicolectomy, sigmoid colectomy/anterior resection, subtotal colectomy, Hartmann’s procedure, or abdominoperineal resection (APR) for colorectal cancer, diverticular disease, or volvulus, with or without colostomy formation, were included. All patients had to be able to give informed consent, and understand the risks and benefits of the study. All patients were seen before surgery in clinic, and given verbal and written information about the design, purpose, risks, and benefits of the study. Written consent was obtained from all study participants, and patients could withdraw themselves from the study at any stage.

#### Exclusion criteria

Patients were excluded if they had an ASA fitness grade of IV or higher, any allergy to serotonin-based medication, active inflammatory bowel disease, moderate-to-severe renal impairment (estimated glomerular filtration rate below 50 ml/min) or severe hepatic impairment, were pregnant, or were receiving preoperative intravenous nutrition (IVN). Patients in whom ileostomy formation was planned were excluded, as were those who had pre-existing gut dysmotility with endocrine, metabolic, or neurological causes. Patients who were unable to consent owing to dementia, cognitive impairment, language difficulties or delirium were not included.

### Demographics and data collection

Demographic data were collected on patient factors including: age, sex, procedure, indication, co-morbidities, and ASA grade. Patients were assessed twice daily (0.800 and 20.00 hours) by a blinded study investigator until they achieved GI-2, defined as passage of stool and tolerance of oral diet^19^, and then daily thereafter. Information on primary and secondary outcomes, blood tests, and complications were recorded on pre-prepared data collection sheets. Patients were telephoned to determine time to passage of stool, if this had not been achieved at the time of discharge. The primary outcome of this study was time to GI-2^19^. Tolerance of oral diet was defined as the ability to eat a solid or semisolid diet of 25 per cent or more of preoperative meal intake without significant nausea or vomiting over two or more consecutive meals. Secondary outcomes recorded included: time until passage of flatus and stool, and tolerance of diet, preoperative and postoperative blood test results from days 1–3, including haemoglobin level, C-reactive protein (CRP) concentration, white cell count (WCC) and differential, renal function, and electrolytes. Duration of hospital stay, need for reoperation, and 30-day readmission rates were also recorded. Volumes or quantities of perioperative and postoperative intravenous fluid, analgesia, and antiemetic and anti-inflammatory medications were recorded. Adverse events were graded by system and severity using the Common Terminology Criteria for Adverse Events version 4 guidelines, and complications using the Clavien–Dindo classification system^20^. As a secondary endpoint, patients were also assessed for PPOI using the definition provided by Vather and colleagues^2^, as well as need for a nasogastric tube (NGT) or IVN. Specifically, patients were diagnosed with PPOI if they met two or more of the following criteria on or after postoperative day 4: nausea or vomiting, inability to tolerate an oral diet over the past 24 h, absence of flatus over the past 24 h, abdominal distension, or radiological evidence of ileus^2^. All patients also completed a Gastroparesis Cardinal Symptom Index (GCSI) questionnaire before surgery and daily until discharge^21^. The GCSI comprises nine patient-reported components, each rated in severity from 0 to 5, with a total score of 45. The questions involve three subscales (postprandial fullness/early satiety, nausea/vomiting, and bloating). Higher scores indicate worse symptoms of gastroparesis.

### Randomization and methods

Patients were randomized by study investigators on the morning of operation to receive either 2 mg prucalopride or 2 mg identical placebo capsule, using a computer-generated randomization list prepared by an external pharmacy. The study medication was prepared by an external pharmacy, block randomized into groups of 10 in a 1 : 1 allocation and numbered sequentially. Participants, study investigators, and clinical staff were all blinded to the allocation. Only the external pharmacy had access to the unblinded study medication allocation data. The study medication was given 2 h before operation, so that prucalopride reached its peak plasma concentration at time of surgery^22^, and continued daily in the morning for up to 6 days, or until the patient had achieved the primary endpoint or was discharged from hospital. All patients undergoing elective colorectal surgery at Auckland City and Dunedin hospitals follow a structured enhanced recovery after surgery (ERAS) protocol. The ERAS protocol included preoperative oral carbohydrate drinks, early introduction of postoperative diet, stepwise analgesia progression, minimization of intravenous fluids, bowel preparation and opiate use, early patient mobilization, subcutaneous thromboprophylaxis, and omission or early removal of drains, lines, NGTs, and catheters.

### Statistical analysis

An *a priori* power calculation was performed using retrospective data from a previous prospective trial^4^ on postoperative ileus at Auckland City Hospital. Pilot data were extracted from 76 patients undergoing elective bowel resection without ileostomy formation, which showed a mean(s.d.) time to GI-2 of 4.9(2.6) days. An α value (chance of a false-positive) of 0.05 was used and a β value of 0.2 (chance of a false-negative), with a study power of 80 per cent. The hypothesis was that prucalopride would reduce the time to GI-2 by a clinically meaningful 25 per cent. Using the computer-simulated bootstrapping technique for power calculation^23^, and a two-tailed test in a 1 : 1 allocation ratio, it was calculated that 65 patients per study arm would be required. An expected 15 per cent drop-out rate was used, for a total recruitment of 150 patients across both study sites.

Statistical analyses were conducted on an intention-to-treat basis and no interim analysis was performed. Data were assessed for normal distribution using a histogram plot and the Shapiro–Wilk test. Normally distributed data were analysed using an independent-samples Student’s *t* test, and data that had an asymmetrical distribution using the Mann–Whitney *U* test. The χ^2^ test was used for categorical univariate analyses. All tests were two-sided and *P* < 0.050 was deemed significant. SPSS^®^ for Mac^®^ version 24 (IBM, Armonk, NY, USA) was used for statistical analysis.

## Results

**Fig. 1:**
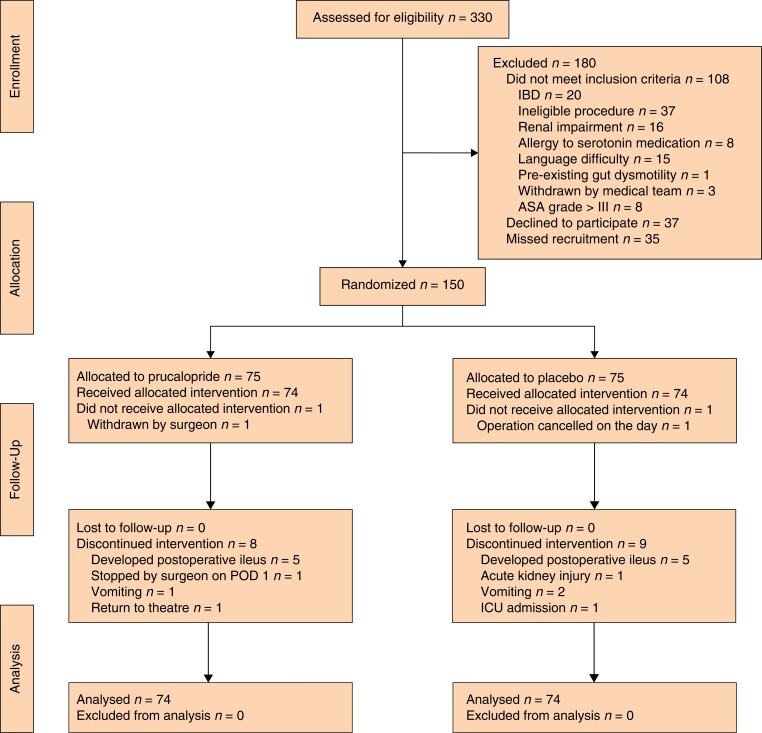
CONSORT diagram for the trial IBD, inflammatory bowel disease; POD, postoperative day.

Some 330 patients were assessed for eligibility, of whom 180 were excluded; 150 patients were randomized, 75 per arm, and one patient in each group was excluded after randomization (*Fig. 1*). One patient in the prucalopride group was withdrawn before operation by the surgeon owing to co-morbidity, and one in the placebo group was withdrawn as their operation was cancelled on the day. Therefore, 148 patients were analysed, 74 per study arm. No patients were lost to follow-up. Eight patients in the prucalopride group and nine in the placebo group discontinued treatment early (11 and 12 per cent respectively), mainly because they developed postoperative ileus. None of the patients who discontinued medication early were excluded from the analysis. Patients received preoperative medication a mean(s.d.) of 138(60) min before surgery, and two patients (1 in each group) did not receive the preoperative dose of study medication.

### Demographic data

The demographics the cohort are described in *Table 1*. Dunedin hospital recruited 91 patients (62 per cent) and Auckland Hospital 57 patients (38 per cent). Demographic data, including age, sex, ethnicity, BMI, ASA grade, and indication for surgery, were not statistically different between groups. The surgical approach (laparoscopic, laparoscopically assisted, open, or converted) was also similar in the two groups. All APRs occurred in the prucalopride group (5 patients); the groups were otherwise similar in terms of type of colonic resection. Perioperative management was similar in the prucalopride and placebo groups. There was no difference in the use of spinal or epidural block: 46 per cent of patients in the prucalopride group had a spinal and 8 per cent an epidural block, compared with 43 and 7 per cent respectively in the placebo group (*P* = 0.870). Of patients who underwent laparoscopic surgery, two had an epidural block (1 in each group). There was no difference in the volume of perioperative crystalloid (*P* = 0.716), units of non-steroidal anti-inflammatory drugs (*P* = 0.882) or units of opiates (*P* = 0.598) between groups.

**Table 1:** Demographic data

	Total (*n* = 148)	Prucalopride (*n* = 74)	Placebo (*n* = 74)
Age (years)*	70.5 (64.5–76.5)	71 (64–78)	69.5 (64–75)
**Sex**			
M	83 (56.1)	45 (61)	38 (51)
F	65 (43.9)	29 (39)	36 (49)
**Ethnicity**			
NZ European	138 (93.2)	68 (92)	70 (95)
Maori	3 (2)	1 (1)	2 (3)
Asian	2 (1.4)	1 (1)	1 (1)
Indian	4 (2.7)	3 (4)	1 (1)
Other	1 (0.7)	1 (1)	0 (0)
BMI (kg/m^2^)*	27 (24.3–31.5)	27.6 (23.7–31.5)	26.8 (23.8–29.8)
**ASA fitness grade**			
I	16 (10.8)	8 (10.8)	8 (10.8)
II	93 (62.8)	43 (58.1)	50 (67.6)
III	39 (26.4)	23 (31.1)	16 (21.6)
**Operation**			
Right hemicolectomy	54 (36.5)	24 (32)	30 (41)
Anterior resection	81 (54.7)	41 (55)	40 (54)
Abdominoperineal resection	5 (3.4)	5 (7)	0 (0)
Subtotal colectomy	8 (5.4)	4 (5)	4 (5)
**Surgical approach**			
Laparoscopic	112 (75.7)	54 (73)	58 (78)
Laparoscopically assisted	13 (8.8)	8 (11)	5 (7)
Open	19 (12.8)	10 (14)	9 (12)
Converted to open	4 (2.7)	2 (3)	2 (1)
**Stoma**			
None	133 (89.9)	66 (89)	67 (91)
Ileostomy	10 (6.8)	3 (4)	7 (10)
Colostomy	5 (3.4)	5 (7)	0 (0)
**Indication**			
Cancer	137 (92.6)	71 (96)	66 (89)
Diverticular disease	10 (6.8)	3 (4)	7 (10)
Volvulus	1 (0.7)	0 (0)	1 (1)
Duration of operation (min)*	188 (142–240)	199 (144–255)	180 (135–226)

Values in parentheses are percentages unless indicated otherwise; *values are median (i.q.r.). NZ, New Zealand.

### Primary outcomes

**Fig. 2:**
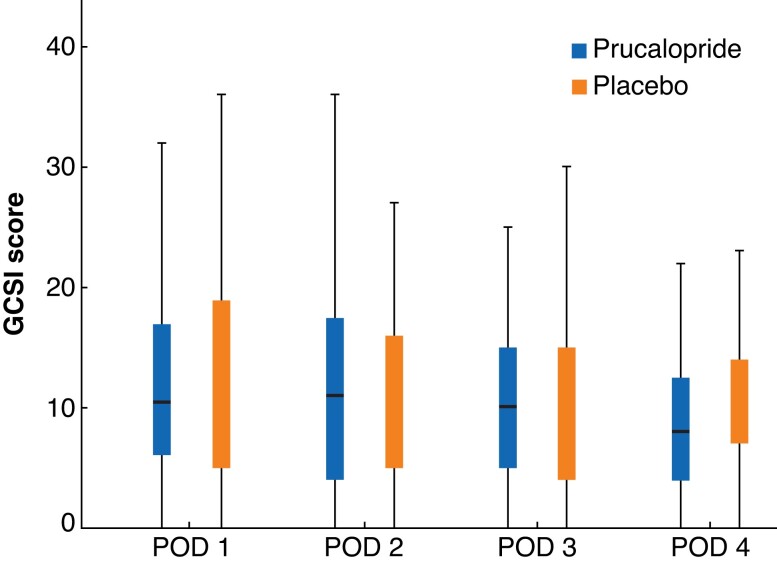
Box plot showing Gastroparesis Cardinal Symptom Index questionnaire scores Median value (bold line), i.q.r. (box), and range (error bars) are shown. GCSI, Gastroparesis Cardinal Symptom Index; POD, postoperative day.

There was no clinically, or statistically, meaningful difference in time to GI-2 (passage of stool and tolerance of diet) between the prucalopride and placebo groups: median 3.5 (i.q.r. 2–5) *versus* 4 (3–5) days respectively (*P* = 0.124). The time to tolerance of oral diet was similar between groups: 2 (0.5–3.5) *versus* 2 (0.5–3.5) days (*P* = 0.669). Patients who received prucalopride had a shorter time to passage of stool than those in the placebo group: 3 (2–4) *versus* 4 (2.7–5.3) days (*P* = 0.027). Median time to flatus was 2 days in both groups, but there was a statistical difference (*P* = 0.029), although this was not considered clinically significant. *Table 2* shows a full statistical analysis of primary and secondary outcomes. The rates of PPOI, NGT insertion, and requirement for IVN were similar in the two groups. Duration of hospital stay was also similar at median 4 (2–6) days in both groups (*P* = 0.929). There was no difference between groups in terms of symptoms reported by patient using the GCSI questionnaire on postoperative days 1 to 4 (*Fig. 2*).

**Table 2:** Results of intention-to-treat analysis

	Total (*n* = 148)	Prucalopride (*n* = 74)	Placebo (*n* = 74)	*P*†
Time to GI-2 (days)*	4 (3–5)	3.5 (2–5)	4 (3–5)	0.124‡
Time to diet (days)*	2 (1–4)	2 (0.5–3.5)	2 (0.5–3.5)	0.669‡
Time to flatus (days)*	2 (1–2)	2 (1.5–2.5)	2 (1.5–2.6)	0.029‡
Time to stool (days)*	3 (2–4)	3 (2–4)	4 (2.7–5.3)	0.027‡
Duration of hospital stay (days)*	4 (3–7)	4 (2–6)	4 (2–6)	0.929‡
**Incidence of PPOI**	32 (21.6)	16 (22)	16 (22)	1.000
**NGT required**	23 (15.5)	10 (14)	13 (18)	0.082
**IVN required**	9 (6.1)	4 (5)	5 (7)	0.785

Values in parentheses are percentages unless indicated otherwise; *values are median (i.q.r.). GI-2, passage of stool and tolerance of oral diet; PPOI, prolonged postoperative ileus; NGT, nasogastric tube; IVN, intravenous nutrition. †χ^2^ test, except ‡Mann–Whitney *U* test.

### Complications and adverse events

Complications and adverse events in the prucalopride and placebo groups are summarized in *Table 3*. The rates of postoperative complications and adverse events were similar. There was no difference between the prucalopride and placebo groups in rates of anastomotic leak (3 *versus* 1 per cent respectively; *P* = 0.560) and reoperation (both 4 per cent; *P* = 1.000), or in readmission rates within 30 days (*P* = 0.597). The rate of cardiac complications was similar (12 *versus* 8 per cent; *P* = 0.414). Patients in the prucalopride group had fewer renal complications (3 *versus* 11 per cent; *P* = 0.049).

**Table 3:** Complications and adverse events

	Total (*n* = 148)	Prucalopride (*n* = 74)	Placebo (*n* = 74)	*P*‡
**Clavien–Dindo grade**				
I	19 (12.8)	9 (12)	10 (14)	0.806
II	31 (20.9)	19 (26)	12 (16)	0.157
III	11 (7.4)	7 (10)	4 (5)	0.347
IV	6 (4.1)	3 (4)	3 (4)	1.000
V	0 (0)	0 (0)	0 (0)	–
**Readmission within 30 days**	16 (10.8)	9 (12)	7 (10)	0.597
**Reoperation**	6 (4.1)	3 (4)	3 (4)	1.000
**Anastomotic leak**	3 (2)	2 (3)	1 (1)	0.560
**CTCAE grade**				
1	39 (26.4)	18 (24)	21 (28)	0.576
2	44 (29.7)	20 (27)	24 (32)	0.472
3	39 (26.4)	22 (30)	17 (23)	0.351
4	7 (4.7)	4 (5)	3 (4)	0.699
5	0 (0)	0 (0)	0 (0)	–
**CTCAE system**				
General	7 (4.7)	6 (8)	1 (1)	0.116
Cardiac*	15 (10.1)	9 (12)	6 (8)	0.414
Gastrointestinal	49 (33.1)	24 (32)	25 (34)	0.861
Infection	18 (12.2)	10 (14)	8 (11)	0.615
Neurological	10 (6.8)	4 (5)	6 (8)	0.512
Renal†	10 (6.8)	2 (3)	8 (11)	0.049
Respiratory	3 (2)	2 (3)	1 (1)	0.560
Vascular	14 (9.5)	7 (10)	7 (10)	1.000
Blood and lymphatic	8 (5.4)	3 (4)	5 (7)	0.467
Injury, poisoning, procedural	4 (2.7)	3 (4)	1 (3)	0.311
Metabolism/nutrition	6 (4.1)	4 (5)	2 (3)	0.405

Values in parentheses are percentages. *Prucalopride group: atrial flutter (1), sinus arrhythmia (1), atrial fibrillation (AF) (5), chest pain (1), sinus tachycardia (1); placebo group: bradycardia (3), AF (1), sinus tachycardia (2). †Prucalopride group: urinary retention (2); placebo group: acute kidney injury (5), urinary retention (3). CTCAE, Common Terminology Criteria for Adverse Events. ‡χ^2^ test.

### Laboratory results

There was no difference in WCC between groups on postoperative days 1 (*P* = 0.465), 2 (*P* = 0.529), and 3 (*P* = 0.860). Postoperative levels of CRP on postoperative days 1 (*P* = 0.544), 2 (*P* = 0.860), and 3 (*P* = 0.725) were also no different.

### Per-protocol analysis

A subgroup, per-protocol, analysis was performed that excluded patients who did not receive preoperative medication (2) and those who had unplanned ileostomy formation (10). The results were consistent with those of the intention-to-treat analysis. Patients in the prucalopride group passed flatus (*P* = 0.041) and stool (*P* = 0.006) faster than those who received placebo, but did not have a shorter time to tolerance of diet (*P* = 0.446) or GI-2 (*P* = 0.177), or a shorter hospital stay (*P* = 0.850). Because all the APRs occurred in the prucalopride group, a further analysis was undertaken to determine whether this had skewed the results. There was no difference in GI-2 (*P* = 0.244), time to tolerance of diet (*P* = 0.419), time to flatus (*P* = 0.062), or duration of hospital stay (*P* = 0.691). However, patients in the prucalopride group had a shorter time to passage of stool (*P* = 0.014).

### Analysis of laparoscopic *versus* open surgery

A *post hoc* analysis was performed including only patients who underwent laparoscopic or laparoscopically assisted surgery (62 in prucalopride group, 63 in placebo group). Patients in the prucalopride group achieved GI-2 a median of 1 day faster than those in the placebo group: 3 (2–4) *versus* 4 (3–5) days (*P* = 0.012). However, there was no difference in median duration of hospital stay: 4 (3–5) *versus* 4 (2.5–5.5) days respectively (*P* = 0.469). The incidence of PPOI (*P* = 0.478), need for NGT insertion (*P* = 0.375), and IVN (*P* = 0.833) were similar in the two groups. There was no difference between groups in postoperative inflammatory markers (WCC or CRP) on days 1–3, or in rates of complications and adverse events, among patients who underwent laparoscopic surgery.

An analysis of patients who had open surgery or conversion to an open procedure (12 prucalopride, 11 placebo) showed no difference in time to passage of flatus (*P* = 0.177) or stool (*P* = 0.687), but there was a trend towards delay to tolerance of diet in the prucalopride group compared with the placebo group: 6.3 (1.5–8.7) *versus* 3 (1.5–4.5) days respectively (*P* = 0.054). Median time to GI-2 was 6.3 (5.1–7.5) days in the prucalopride group *versus* 5 (3–7) days in the placebo group (*P* = 0.204). There was no difference between prucalopride and placebo groups in duration of hospital stay (*P* = 0.147), or in complications or adverse events, among those who had open surgery.

## Discussion

This randomized placebo-controlled double-blind trial found that, overall, prucalopride did not improve time to GI-2 or reduce the rate of PPOI compared with placebo in patients undergoing elective colorectal surgery. Although prucalopride improved time to passage of flatus and stool, there was no improvement in time to tolerance of diet or symptoms of gastroparesis. Prucalopride did not shorten the postoperative hospital stay, but was found to be safe and did not increase cardiac or anastomotic complications.

A possible advantage of prucalopride was noted in the laparoscopic surgery cohort. Prucalopride significantly improved time to GI-2 by 1 day among patients who underwent laparoscopic or laparoscopically assisted surgery. This is a clinically meaningful reduction in time to recovery of gut function, but it did not translate to a difference in duration of hospital stay. It is important to note that this was a *post hoc* analysis and not a part of the original statistical analysis plan. However, most patients in this study underwent laparoscopic surgery (84.5 per cent), in adequate numbers to suggest that these results may be meaningful. Inflammation plays a key role in the development and prolongation of postoperative ileus^9,11^. The inflammatory response after laparoscopic surgery is significantly less than that after open surgery^24,25^. It is, therefore, possible that prucalopride was ineffective in patients who underwent open surgery owing to the marked increase in inflammatory response to laparotomy. Further studies assessing the differences in postoperative inflammatory markers are planned.

The strength of this study is that it is a large RCT comparing prucalopride with placebo in patients undergoing elective colorectal surgery. Giving prucalopride before and after surgery is a novel feature of this work. Preoperative administration is important so that prucalopride is active at time of surgery, which is when the neurally and inflammatory-mediated mechanisms of ileus commence^11^.

There are some limitations to the present study. Although there were differences in ileostomy and APR rates between groups, the authors do not believe these would have affected the primary outcomes, as they were accounted for in subgroup analysis. All APRs occurred in the prucalopride group, but there were few such procedures in this cohort. The number of patients who had an unplanned ileostomy was also low in both groups, and fell below the study’s drop-out rate of 15 per cent. The results of the per-protocol analysis (with ileostomy excluded) were consistent with those of the primary analysis.

One consideration is whether an adequate dose of preoperative prucalopride was provided. Stakenborg and colleagues^12^ reported a reduction in postoperative interleukin (IL) 6, IL-8 and tumour necrosis factor α levels in intestinal samples from patients who received 4 mg preoperative prucalopride, but no difference in serum samples. However, increasing the prucalopride dose from 2 to 4 mg did not significantly improve outcomes for patients with chronic constipation^26,27^, and both 2 and 4 mg prucalopride were sufficient to improve gastric emptying times in patients with gastroparesis in recent series^15,28^. It is unclear whether patients would benefit from additional preoperative dosing to fully benefit from the anti-inflammatory properties of prucalopride, or to achieve a steadier state of the drug before operation.

Postoperative ileus remains a significant problem for patients and healthcare professionals after colorectal surgery. Although prucalopride was ineffective in improving time to GI-2 and reducing the rate of PPOI in the overall cohort, it had an apparent advantage in time to passage of stool for all patients, and significantly improved time to GI-2 in patients undergoing laparoscopic surgery. Prucalopride may therefore be effective in improving time to return of postoperative gut function in selected patients undergoing elective minimally invasive colorectal surgery.
